# A Machine Learning Approach to Qualitatively Evaluate Different Granulation Phases by Acoustic Emissions

**DOI:** 10.3390/pharmaceutics15082153

**Published:** 2023-08-17

**Authors:** Ruwen Fulek, Selina Ramm, Christian Kiera, Miriam Pein-Hackelbusch, Ulrich Odefey

**Affiliations:** 1 Department of Life Science Technologies, OWL University of Applied Sciences and Arts, Campusallee 12, 32657 Lemgo, Germany; ruwen.fulek@th-owl.de (R.F.);; 2 Department of Electrical Engineering and Computer Science, OWL University of Applied Sciences and Arts, Campusallee 12, 32657 Lemgo, Germany; 3 PHARBIL Pharma GmbH, Reichenberger Str. 43, 33605 Bielefeld, Germany

**Keywords:** wet granulation, acoustic classification, machine learning, convolutional neural networks

## Abstract

Wet granulation is a frequent process in the pharmaceutical industry. As a starting point for numerous dosage forms, the quality of the granulation not only affects subsequent production steps but also impacts the quality of the final product. It is thus crucial and economical to monitor this operation thoroughly. Here, we report on identifying different phases of a granulation process using a machine learning approach. The phases reflect the water content which, in turn, influences the processability and quality of the granule mass. We used two kinds of microphones and an acceleration sensor to capture acoustic emissions and vibrations. We trained convolutional neural networks (CNNs) to classify the different phases using transformed sound recordings as the input. We achieved a classification accuracy of up to 90% using vibrational data and an accuracy of up to 97% using the audible microphone data. Our results indicate the suitability of using audible sound and machine learning to monitor pharmaceutical processes. Moreover, since recording acoustic emissions is contactless, it readily complies with legal regulations and presents Good Manufacturing Practices.

## 1. Introduction

Granulation is a commonly applied process in pharmaceutical production. The quality of resulting granules thereby directly depends on material- and process-related parameters. Many studies and developments thus aim for steering these processes towards pre-defined quality attributes of granules, such as a targeted granulation size and distribution, or their moisture content [1,2].

Processes can be tracked by analyzing the quality of intermediates and products offline/atline. According to the FDA PAT [3] initiative, however, inline monitoring should be favored. Common proxy variables included in this regard power consumption of the impeller, electrical quantities of the granule mass like capacitance and resistance, microwave measurements, near-infrared spectroscopy analysis, particle size measurements, stress and vibration, as well as acoustic emissions [4]. It should be noted, however, that inline probes extending into the process areas can influence the processes themselves, which is why they cannot be retrofitted after process approval has been granted.

Utilizing acoustic emissions of the process, which can be recorded contactless by microphones—which themselves can easily be retrofitted to a process and does not protrude into a process area—thus seem to be a promising PAT approach. With a view on the high shear wet granulation process we will focus on in our present study, the literature particularly mentions that acoustic signals emitted during a granulation process can provide material- and process-related information [5].

Furthermore, in fact, the literature provides approaches that aim to relate information contained in the acoustic signals to the physical properties of the granule mass or to characterize the granulation process itself. The physical properties of interest are the density of the blend, its liquid content, the particle size distribution, its compression behavior, and many more. Regarding granulation processes, studies were aimed at characterizing different process stages as well as detecting the end-point of the process [6,7]. Emissions from the ultrasonic range have been able to describe flow dynamics in fluidized-bed granulations [8]. Emissions from the acoustic spectrum characterized the different stages of a high-shear granulation process [7,9,10]. For instance, the mean frequency of an acoustic signal indicated the end-point of a high-shear granulation process for granulators of different sizes [7]. The power spectral density of the acoustic signal also contains information about critical quality attributes, like over-wetting [11]. However, the particular literature is rather old and, although the process assessment was successful for single processes, it was based on a subjective choice of evaluation criteria, which only correspond to each individual setup. Improved classification rates and abandonment of subjective selection of evaluation criteria can be expected from machine learning approaches. However, the use of machine learning for granulation process monitoring and endpoint prediction by acoustic emission has not yet been addressed in the field of pharmaceutics—and only rarely for pharmaceutical granulation processes in general [12].

In our study, we will use a neural network to classify different stages of a high shear wet granulation process by acoustic emissions. As convolutional neural networks (CNNs) [13] are the state of the art for classify environments from the sounds they produce [14], we will focus our research on these networks. We are going to follow a supervised learning approach to classify and recognize the granulation process according to the quality of the granule mass. This targeted quality directly corresponds to the amount of liquid and the related compression density of the granule mass [1].

## 2. Materials and Methods

First, this section reports the experimental setup and summarizes the materials used. Second, we describe the process of data acquisition and data preprocessing. Section 2.5 details the used machine learning approach.

### 2.1. Experimental Setup

**Figure 1:**
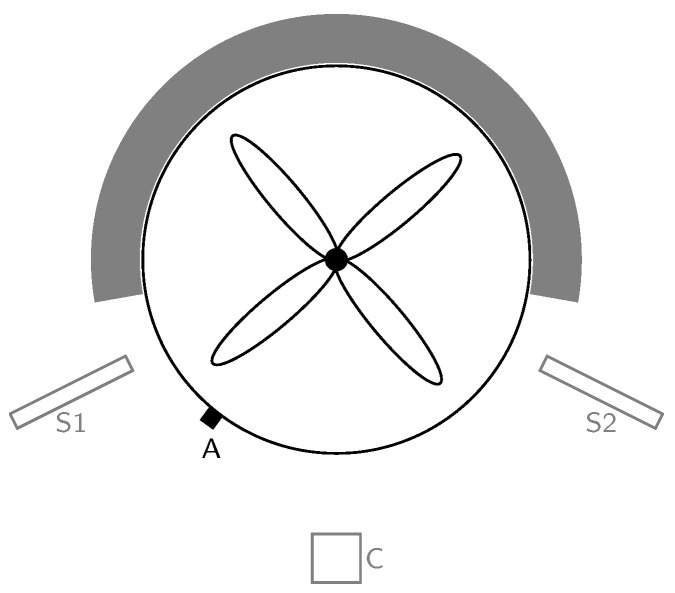
Top view of granulator and sensor setup. A is the position of the acceleration sensor. S1 and S2 are the positions of the shotgun microphones. C is the position of the single condenser microphone. The gray area illustrates the casing of the mixing system.

We used a laboratory-scale mixing system to perform a wet granulation process. Different sensors monitored and recorded the acoustics of the process. Figure 1 sketches the position of the sensors relative to the mixing system.

For granulation, we used a Thermomix${}^{®}$ TM6 (Vorwerk, Wuppertal, Germany). To mimic the behavior of an industrial intensive mixer, we replaced the original agitator by a custom-made mixing knife. This modification ensured proper material mixing, like in a Diosna P1-6 intensive mixer.

A peristaltic pump (Petro Gas 1B.1003-r/65) added water at a defined rate. In addition, a laboratory scale (PCE Instruments PCE-BS 3000) controlled the amount of water added.

The laboratory environment was open but quiet, there was no air-conditioning.

### 2.2. Granulation Process

We used a binary placebo formulation of pharmaceutical powder materials for the granulation process. In particular, we used Lactose Monohydrate (GranuLac${}^{®}$ 200, Meggle, Wasserburg, Germany) and Microcrystalline Cellulose (VIVAPUR${}^{®}$ 101, JRS Pharma, Rosenberg, Germany). For all experiments, the ratio of Lactose Monohydrate to Microcrystalline Cellulose was 80 to 20. The granulation process was performed with a total dry mass of 150 g, as described in Reference [1]. Water was added with 2 mL min^−1^.

Each of the 10 granulation runs took 75 min to complete, and was divided into three phases. These three phases were defined on the basis of granule properties, related to their moisture content. We have recently shown that, for the given binary placebo formulation, a moisture content on dry basis of 33 percent (25 percent on wet basis) gives optimum material properties for processing [1]. According to that, we defined the granulation phase containing the optimum amount of water as *opt*. The granulation phase containing less water than the optimum is called *dry*. The granulation phase containing more water than the optimum is called *wet*. In the first phase (*dry*), water was continuously added for the first 25 min until the moisture content on dry basis reached 33 percent, i.e., until 50 g of water has been added.

In the second phase (*opt*), mixing was carried out for 25 min without adding water. In the third phase (*wet*), water was added for 3.5 min to achieve a moisture content on dry basis of 38 percent, i.e., 7 g of water was added. After that, mixing was continued for another 25 min. We chose time intervals of equal length both to obtain sufficient data and to ensure that the data sets are evenly distributed throughout the phases.

Table 1 lists all phases, the corresponding timing information, and the details about water addition during each phase.

### 2.3. Data Acquisition

We recorded the acoustics and vibrations using various sensors for the complete 75 min of each run. We tried different sensors for sound recording: a simple condenser microphone (Rode NT-USB), two shotgun microphones (the t.bone EM 9600), and an acceleration sensor (type 4508-B, Brüel & Kjær). The single condenser microphone was placed right in front of the mixing bowl. The shotgun microphones were placed on the left and right side of the mixing bowl with a distance of approximately two centimeters. The acceleration sensor was attached to the mixing bowl using epoxy. It captured the vibrations of the mixing bowl and thus no audible sound signal. We used Audacity${}^{®}$ v3.1.3 (www.audacityteam.org accessed on 8 July 2023), to record signals from all sensors. The sampling rate of the recordings was 44.1 kHz. The audio files were stored in lossless FLAC format (www.xiph.org/flac accessed on 8 July 2023). In addition, Audacity${}^{®}$ allowed for labeling of the acoustic recordings to indicate the different phases of the granulation process. This labeling is mandatory for supervised machine learning.

### 2.4. Data Preparation and Processing

In the first step, the audio data were converted into the time-frequency domain using a short-time Fourier transformation [15]. We chose a window size of $n_{\mathrm{fft}}=2048$ and a hop length of $n_{\mathrm{hop}}=512$. Next, we transformed the Fourier frequencies into the mel scale [16]. The mel scale has been used previously to extract features of audio signals for acoustic scene classification [16,17,18]. Here, we chose $n_{\mathrm{mel}}=128$ as the number of mel frequency coefficients. Then, we split the mel spectrograms into non-overlapping windows, each containing 32 columns. In the final step, each window was log-scaled and, therefore, we ended up with log-mel spectrograms with a size of 128 × 32. These windows of log-mel spectrograms are denoted as samples and are the input to our neural network. We used the Python library librosa [19] for data preparation and processing.

### 2.5. Machine Learning Setup

To classify the different phases of a mixing process, we trained CNNs [13] using a supervised learning approach. The acceleration sensor, the single condenser microphone, and the double shotgun microphone data each were used to train separate CNNs. During the training process, the network uses the samples from the sound recordings and their annotations of the corresponding granulation phases. Then the network can learn how sounds/features are related to each of these phases. CNNs automatically derive learning features from the available data. When dealing with large-scale data input, this automatism is valuable. Relevant data characteristics do not have to be manually designed or extracted, but are a direct result of the learning process. Here, we used a VGG-style (visual geometry group) network as proposed in [20]. Table 2 summarizes the network architecture we used in this study.

In short, the input layer iteratively receives batches containing samples of the computed log-mel spectrograms. The four convolutional layers construct feature maps from the input with decreasing complexity. Each convolutional layer uses batch normalization and ReLU activation before sending its results to the next layer. Two fully-connected layers are applied before the last layer returns a classification probability. The output shows the CNN’s classification probability for each of the three granulation phases. Simply put, a high classification accuracy indicates that the network is able to identify the different states of the granulation process, based on short acoustic profiles. For readers unfamiliar with machine learning, we emphasize that the network distinguishes only the three states defined beforehand. Discrimination between further states needs re-labeling of the data and re-training of the network.

Training of the CNN was performed via gradient descent using the Adam optimizer with the parameters $\beta_{1}=0.9$ and $\beta_{2}=0.999$. We set the learning rate to $10^{-3}$. The loss function was binary cross-entropy. Training was stopped automatically when validation did not improve the learning outcome for twenty consecutive learning epochs. The training batch size was 128. We implemented all networks, analyses, and visualizations in the programming language Python. In particular, we used the libraries NumPy [21], Tensorflow [22], and matplotlib [23]. A training procedure usually took between 20 and 40 min using GPU acceleration on an NVIDIA Quadro P4000.

### 2.6. Train, Validation and Test Sets

To determine the networks generalizability and a sufficient robustness to small environmental changes, like differences in room temperature or air humidity, the available audio data has to be properly split into a training set and a test set. The test set is only used after the training process. To meet these criteria in the best possible way, we decided to make the training/test splits between the recordings and, depending on the experiment (cf. Section 3.2), between the individual days. We did not perform training/test splits within a single recording. During the training process, a part of the training set is split off into a validation set. Here, we chose a common ratio of 80:20. The main purpose of the validation set is to have a metric that shows if there is still improvement in the learning procedure. The validation set must not be confused with the test set.

## 3. Results and Discussion

We report on the performance of correctly classifying the state of a granulation process in this section. We first show the results of our conducted granulation procedures. Then, we show our derived training/test setups, and the classification accuracy of the individual trained CNNs and their corresponding microphones.

### 3.1. Granulation Results

**Figure 2:**
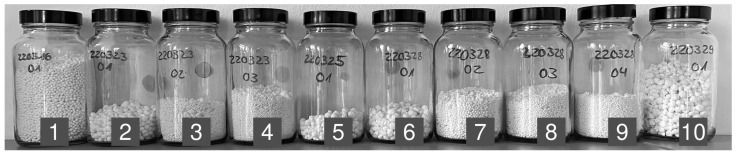
Comparison of the granules that resulted out of the granulation. The top number on each container gives the date of the experiment in yymmdd format. The lower number indicates the charge of the given day.

In total, we performed ten granulation experiments. Figure 2 shows the obtained granules for each run.

Here, one observes that the granules of the first charge of each day are significantly larger than those in the remaining containers. We attribute this difference in size to the heating up of the mixing system during the first run. In practice, one wants to avoid granules of too large a size. Consequently, the five initial charges represent non-optimal mixing and granulation behavior. Our developed classifier also recognizes this possibility, cf. Section 3.6.

### 3.2. Training/Testing Setups Based on Granulation Results

For our analyses, we choose the recordings from the experiments that resulted in granules of a small size. These recordings are container 3 and 4 from March 23, and containers 7–9 from March 28, cf. Figure 2. Each of these five recordings have temporal labels according to the defined states *dry*, *opt*, and *wet*. Out of this data pool, we construct three training/testing setups.

1. Multi-day training: Two recordings from each day (containers 3–4, 7–8) represent training and validation data, the remaining recording (container 9) is used for testing. This setup provides the largest amount of data and follows the general mantra that the more data available, the better;
2. Single-day training: All recordings from March 28 (containers 7–9) represent training and validation data, the recordings from March 23 (containers 3–4) are used for testing. This setup follows a strict separation of training and test data between the days, and thus gives a better impression of generalizability. We note that this is potentially the most practical use case;
3. Little data: Only one recording from March 23 (container 3) is used for training and validation, the recordings from March 28 (containers 7–9) are used for testing. Using this setup, we extend the second approach with an additional investigation of the impact of little training data on the classification accuracy.

### 3.3. Overall Classification Accuracy

To assess the performance of the CNN, we look at the classification accuracy. The classification accuracy is the ratio of the number of correctly identified samples to the total number of samples. Table 3 shows the classification accuracy for all training/test setups as described in Section 3.2. A distinction is made between the sensors and their corresponding networks.

All sensor data yield a classification accuracy of around 90 percent and above for both the multi-day and single-day setups. The multi-day training setup generally gives the highest classification accuracy. The single condenser microphone yields the highest score of 97.1 percent, followed closely by the two shotgun microphones, with 96.7 percent.

The performance drops slightly if the CNN tries to classify data from one day, but learns to identify the individual process states with data from a different day (single-day training setup). Here, the condenser microphone data gives again the highest performance score of 95.2 percent. Classification accuracy using shotgun microphone data is now at 91.9 percent.

When the training data is limited to one recording and the test set is from a different day (little data setup), the classification accuracy for the acceleration sensor and condenser microphone setups drops below 60 percent. The double shotgun microphones perform best in this setup, with an accuracy of 82.4 percent.

Data from the acceleration sensor give the lowest performance score of all sensors, independent of the training/testing setup. This result is surprising, since we expected the different granulation states to suitably translate into vibrations of the mixing bowl. However, a classification accuracy of about 90 percent is still a remarkable result.

### 3.4. Time-Varying Classification of Granulation Phases

**Figure 3:**
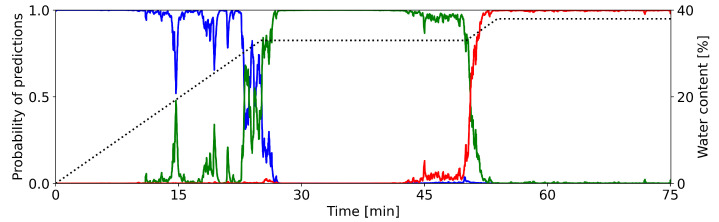
Predictions of granulation states as calculated by the CNN (condenser microphone recording of container 9, cf. Figure 2). Colored lines show the classification probabilities of the CNN (**left** y-axis): blue = *dry*, green = *optimal*, red = *wet*. The dotted line shows the water content (**right** y-axis). The graph is smoothed out with a central moving average of k=10 for each direction.

In this section, we want to take a closer look at the classification certainties of the CNNs, i.e., how sure is the CNN when classifying a sample during the granulation process. Figure 3 shows the networks classification outputs of the single condenser microphone data from the multi-day training setup. The colored lines show the probability that the process is in a particular state. Blue, green, and red indicate the states *dry*, *optimal*, and *wet*, respectively. The dotted line shows the water content.

During each phase of the granulation process, the CNN properly recognizes the current state. During the first 20–22 min, the network gives the highest probability to state *dry*. The probability for state *wet* is essentially zero. The CNN occasionally attributes some considerable probability to the state *optimal*.

Between 22 and 27 min, the predictions of the CNN show a distinct change from state *dry* to *optimal*. The decreasing value of the blue line and the increasing value of the green line indicate this change. Here, one can observe an irregular progression from one state prediction to the next. This behavior is in contrast to the change observed later. Until minute 50, the network dominantly predicts the process to be in state *optimal*, it attributes almost no probability to the other states.

Again, we see a clear change after around 50 min. Now, the probability for state *optimal* drops rather sharply to zero, while the probability for state *wet* increases likewise. This progression is more regular, as compared to the earlier change in states. For the remaining time of the process, the predictions of the network do not change. It attributes almost all probability to state *wet*, while states *dry* and *optimal* are not predicted.

In summary, the designed CNN is able to correctly classify the defined phases of a granulation process. The calculated probabilities clearly indicate the individual time intervals of each phase. They also bring out the progression from one state to the next.

### 3.5. Classification Accuracy for Individual Phases

Figure 3 already gave a qualitative description for the ability to predict individual states of the granulation process.

**Figure 4:**
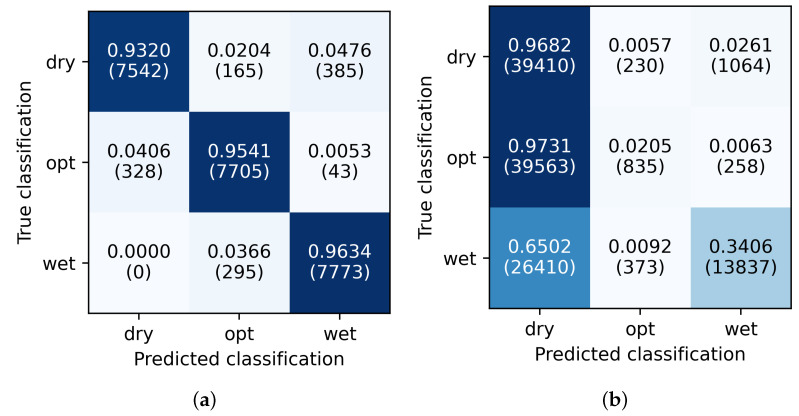
Confusion matrices of the samples classified by the neural networks trained in the multi-day training setup. The numbers are accumulated for all devices/networks. Subfigure (**a**) is the confusion matrix of its test set (container 9, cf. Figure 2). Subfigure (**b**) is the confusion matrix for all recordings of the granulation processes, where the granules do not have the desired optical properties (container 1–2, 5–6, 10, cf. Figure 2). We show absolute numbers in parentheses to indicate the counts of each prediction, to emphasize the reliability of the CNNs over thousands of samples.

The ability to identify individual states is usually shown in a confusion matrix. Here, one simply counts how often the network predicts a possible state, given one of the true states. The confusion matrix of a perfect classification has ones on the diagonal and zeros elsewhere. Figure 4a shows the confusion matrix for the samples from the multi-day training setup. The values on the diagonal are well above 90 percent. A total of 4.8 percent of the samples with true state *dry* are misclassified as class *wet*. Upon closer examination, it turns out that the misclassifications exclusively occur on the acceleration sensor data. On the other hand, no samples of the class *wet* are missclassified as *dry*. The rest of the misclassifications appear between consecutive phases.

### 3.6. Classification Behavior on Improper Granulation Processes

In Figure 4b, we include the confusion matrix for a further testing setup. Here, we assess the CNNs classification behavior on recordings from processes giving improper granule size.

First, the CNNs are able to correctly predict the state *dry*. Given the true state *opt*, the CNNs misclassify it as the state *dry* in more than 97 percent of the cases. The fraction of correct predictions for the state *wet* is around 35 percent, significantly lower compared to the other training/test setups.

At first sight, these misclassifications seem to be undesired; however, at second thought, this behavior simply reflects that the acoustics emitted by the improper granulation processes are always more similar to *dry* or *wet*. The features extracted in the training process for the *opt* state must be on a narrow degree between the other two states. After all, the network recognizes improper states, i.e., granules of too large a size.

### 3.7. Time-Varying Classification of Granulation Phases Giving Improper Results

**Figure 5:**
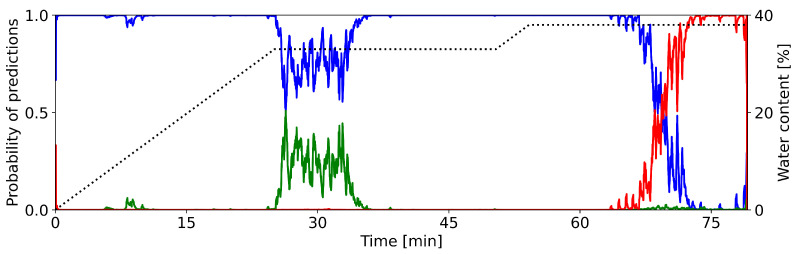
Predictions of granulation states as calculated by the CNN (condenser microphone recording of container 2, cf. Figure 2). Colored lines show the classification probabilities of the CNN (**left** y-axis): blue = *dry*, green = *optimal*, red = *wet*. The dotted line shows the water content (**right** y-axis). The graph is smoothed out with a central moving average of k=10 for each direction.

Figure 5 underscores the findings just described.

It shows the temporal course of the predictions of the network. Here, only two distinct phases are detectable. The first phase lasts from 0 to 65 min. Here, the network predicts the granulation process only to be in state *dry*. There is a short period between 25 and 35 min, during which the state *optimal* also has considerable prediction probability. However, this interval lasts only for about ten minutes, then prediction is exclusively for state *dry*.

At around 65 min, the network starts having more and more confidence in the prediction of state *wet*. This prediction then takes over for the remaining time of the granulation process.

In summary, the CNNs trained with samples from proper granulations are also capable of distinguishing improper developments in the granulation process. In our opinion, this behavior is the most relevant aspect for industrial applicability of the presented approach.

## 4. Conclusions and Outlook

In this study, we recorded acoustics and vibrations from a three-phase granulation process and investigated the effectiveness of machine learning in discriminating between the different phases. Using CNNs trained with log-mel spectrograms as input, our aim was to classify the phases of the the granulation process based on the quality of the granule mass. We were able to reliably predict the different phases for all setups, with a classification accuracy of almost always above 90 percent, provided that the train dataset contained at least two recordings. Reducing the amount of data for training to one recording lowered the classification performance, underscoring the common machine learning mantra that training data should be as numerous as possible. Testing the CNN with data from improper granulation processes, i.e., processes yielding granules that were too large, led to correct misclassifications. These results underline the CNN’s ability to distinguish between good and bad granulation results with a high degree of certainty. Besides providing evidence of the suitability of machine intelligence in discriminating between different phases, it is important to highlight additional advantages. By utilizing the entire spectrum for training the CNNs, this approach offers the benefit of eliminating the subjective selection of evaluation criteria. Beyond our study, a continuing research task is to record acoustic emissions in an industrial pharmaceutical production environment, and to verify whether the methodology is transferable to a larger scale. In addition, it should be investigated whether the methodology can be used for quality management of slightly modified formulations for which audio data has not been previously collected. Finally, the methodology could be further developed for real-time endpoint detection.

## Figures and Tables

**Table 1:** Division of the granulation process into three phases with associated timing and water addition specifications.

Phase	Timing [min]	Information on Moisture Content ω on Dry Basis
*dry*	0–25	Water addition from 0 to 25 min until ω=33%.
*opt*	25–50	No water addition; ω remains at 33%.
*wet*	50–75	Water addition from 50 to 57:30 min until ω=38%

**Table 2:** CNN architecture of this study: The data input layer is followed by four convolutional layers. All convolutions have zero padding and are centered (stride = 1). Then, two fully-connected layers are next. Dropout refers to the fraction of neurons randomly left out in each learning step. We also include kernel regularization for these layers. The output layer represents the classification probability obtained via a SoftMax function.

Layer Name	Settings and Operations
Input	128 × 32 × 1
Conv1	3 × 3 Conv×64c-BN-ReLU
	3 × 3 Conv×64c-BN-ReLU
	2 × 2 MaxPooling
Conv2	3 × 3 Conv×128c-BN-ReLU
	3 × 3 Conv×128c-BN-ReLU
	2 × 2 MaxPooling
Conv3	3 × 3 Conv×256c-BN-ReLU
	3 × 3 Conv×256c-BN-ReLU
	2 × 2 MaxPooling
Conv4	3 × 3 Conv×512c-BN-ReLU
	3 × 3 Conv×512c-BN-ReLU
	2 × 2 MaxPooling
FC1	Dense (# of units = 1024, activation = ReLU)
	Dropout (*p* = 0.5)
FC2	Dense (# of units = 1024, activation = ReLU)
	Dropout (*p* = 0.5)
	Dense (# of units = 3)
	GlobalAveragePooling
Output	3-way SoftMax

**Table 3:** Test accuracy of sound recording devices and their corresponding CNN for the training/test setups.

**Train/Test Setup as** **Described in Section 3.2**	Classification Accuracy [%] for Different Sensors and Setup		
	**Acceleration Sensor**	**Condenser Microphone**	**Two Shotgun Microphones**
1. Multi-day training	89.5	97.1	96.7
2. Single-day training	89.0	95.2	91.9
3. Little data	58.4	59.5	82.4

## Data Availability

Not applicable.

## References

[1] Ramm S., Fulek R., Eberle V.A., Kiera C., Odefey U., Pein-Hackelbusch M. (2022). Compression Density as an Alternative to Identify an Optimal Moisture Content for High Shear Wet Granulation as an Initial Step for Spheronisation. Pharmaceutics.

[2] Reimers T., Thies J., Stöckel P., Dietrich S., Pein-Hackelbusch M., Quodbach J. (2019). Implementation of real-time and in-line feedback control for a fluid bed granulation process. Int. J. Pharm..

[3] U.S. Food and Drug Administration, U.F.D (2023). Current Good Manufacturing Practice Regulations. https://www.fda.gov/drugs/pharmaceutical-quality-resources/current-good-manufacturing-practice-cgmp-regulations.

[4] Hansuld E., Briens L. (2014). A review of monitoring methods for pharmaceutical wet granulation. Int. J. Pharm..

[5] Liu B., Wang J., Zeng J., Zhao L., Wang Y., Feng Y., Du R. (2021). A review of high shear wet granulation for better process understanding, control and product development. Powder Technol..

[6] Whitaker M., Baker G., Westrup J., Goulding P., Belchamber R., Collins M. (2000). Applications of acoustic emission to the monitoring and end point determination of a high shear granulation process. Int. J. Pharm..

[7] Briens L., Daniher D., Tallevi A. (2007). Monitoring high-shear granulation using sound and vibration measurements. Int. J. Pharm..

[8] Tsujimoto H., Yokoyama T., Huang C., Sekiguchi I. (2000). Monitoring particle fluidization in a fluidized bed granulator with an acoustic emission sensor. Powder Technol..

[9] Daniher D., Briens L., Tallevi A. (2008). End-point detection in high-shear granulation using sound and vibration signal analysis. Powder Technol..

[10] Hansuld E.M., Briens L., McCann J.A.B., Sayani A. (2009). Audible acoustics in high-shear wet granulation: Application of frequency filtering. Int. J. Pharm..

[11] Hansuld E., Briens L., Sayani A., McCann J. (2012). Monitoring quality attributes for high-shear wet granulation with audible acoustic emissions. Powder Technol..

[12] Lou H., Lian B., Hageman M.J. (2021). Applications of Machine Learning in Solid Oral Dosage Form Development. J. Pharm. Sci..

[13] LeCun Y., Bengio Y., Hinton G. (2015). Deep Learning. Nature.

[14] Lagrange M., Mesaros A., Pellegrini T., Richard G., Serizel R., Stowell D. (2022). In Proceedings of the 7th Workshop on Detection and Classification of Acoustic Scenes and Events (DCASE 2022).

[15] Smith S.W. (2003). Digital Signal Processing: A Practical Guide for Engineers and Scientists.

[16] Qu Y., Li X., Qin Z. (2022). Acoustic scene classification based on three-dimensional multi-channel feature-correlated deep learning networks. Sci. Rep..

[17] Mesaros A., Heittola T., Virtanen T. Acoustic Scene Classification: An Overview of Dcase 2017 Challenge Entries. Proceedings of the 2018 16th International Workshop on Acoustic Signal Enhancement (IWAENC).

[18] Zhang T., Feng G., Liang J., An T. (2021). Acoustic scene classification based on Mel spectrogram decomposition and model merging. Appl. Acoust..

[19] McFee B., Raffel C., Liang D., Ellis D.P., McVicar M., Battenberg E., Nieto O. librosa: Audio and music signal analysis in python. Proceedings of the 14th Python in Science Conference.

[20] Simonyan K., Zisserman A. Very deep convolutional networks for large-scale image recognition. Proceedings of the International Conference on Learning Representations.

[21] Harris C.R., Millman K.J., van der Walt S.J., Gommers R., Virtanen P., Cournapeau D., Wieser E., Taylor J., Berg S., Smith N.J. (2020). Array programming with NumPy. Nature.

[22] Abadi M., Agarwal A., Barham P., Brevdo E., Chen Z., Citro C., Corrado G.S., Davis A., Dean J., Devin M. (2015). TensorFlow: Large-Scale Machine Learning on Heterogeneous Systems. Software. tensorflow.org.

[23] Hunter J.D. (2007). Matplotlib: A 2D graphics environment. Comput. Sci. Eng..

